# Out-of-pocket healthcare expenditures in older Mexican people based on their social security status

**DOI:** 10.1093/heapol/czaf103

**Published:** 2025-12-03

**Authors:** Guillermo Salinas Escudero, Carmen García Peña, Héctor García Hernández

**Affiliations:** Center for Economic and Social Studies in Health, Hospital Infantil de México Federico Gómez, Dr. Márquez Street 162, Doctores, Cuauhtémoc, 06720, Mexico City, Mexico; Head of the National Institute of Geriatrics, Anillo Perif. 2767, San Jerónimo Lídice, La Magdalena Contreras, 10200, Mexico City, Mexico; Health Research Division, National Institute of Geriatrics, Anillo Perif. 2767, San Jerónimo Lídice, La Magdalena Contreras, 10200, Mexico City, Mexico; Facultad de Medicina, Universidad Nacional Autónoma de México, Escolar 411A, Copilco Universidad, Coyoacán, 04360, Mexico City, Mexico

**Keywords:** health systems, social security, health insurance, health expenditures, aged

## Abstract

Out-of-pocket health expenditures (OOPEs) represent a financial strain that can increase the risk of impoverishment, especially in older people. Universal health coverage is the primary strategy to ensure financial protection. The Mexican health system is based on social security. Therefore, the objective of this research is to analyze the relationship between OOPEs and social security status over time among Mexican adults aged 50 and older. A secondary analysis was made using data from the 2012, 2015, 2018, and 2021 waves of the Mexican Health and Aging Study. Multivariable linear regression models were performed to identify the relation between social security and OOPE. Individuals without social security reported the lowest mean expenditures. In contrast, older people with social security stability showed a steady increase in spending throughout the period, reporting the highest mean expenditures on total OOPE. Other variables, such as education, work, economic situation, multimorbidity, disability, and self-rated health status, show a greater relation with OOPE in contrast with social security. Our findings indicate that older adults with stable social security coverage reported the highest OOPE. This finding contrasts with international evidence on the protective role of health insurance. These findings may be attributed to four factors: (i) the challenging epidemiological profile of older adults characterized by chronic diseases and disability, (ii) the structural and organizational changes in the Mexican health system following the political transition in 2018, (iii) a decline in healthcare access among older adults during the COVID-19 (coronavirus disease) outbreak, and (iv) the longstanding oversaturation and low health resources in the health system.

Key messages

- Out-of-pocket health expenditures (OOPEs) can increase the risk of impoverishment.
- Medicines are the largest component of OOPE. However, hospitalizations represent the highest expense.
- Older people with social security (SS) have the highest mean expenditures on OOPE.
- Education, economic situation, morbidity, and disability relate more to OOPE than SS.
- Our finding contrasts with evidence on the protective role of health insurance.

## Introduction

Older adults are a demographic group expected to grow steadily worldwide (United Nations Department of Economic and Social Affairs, Population Division 2022). Their epidemiological profile is mainly characterized by chronic conditions and functional limitations (Prince *et al.* 2015), which often demand access to healthcare services. In this context, limited access may contribute to delays in diagnosis and treatment, potentially increasing the risk of disability, death, and higher healthcare costs (González-Block *et al.* 2021).

One of the main objectives of health systems is to ensure financial protection (Murray and Frenk 2000), which implies that individuals are not forced to delay or forgo necessary medical care due to economic constraints and that households are not exposed to out-of-pocket health expenditures (OOPEs) that compel them to deplete savings, incur debt, or sacrifice the consumption of other essential goods and services. Such financial strain can ultimately increase the risk of impoverishment (Sesma-Vázquez *et al.* 2005).

Universal health coverage is the primary strategy through which governments seek to reduce OOPE (Dong 2009). Achieving this objective requires a health system that ensures comprehensive population coverage (Jalali *et al.* 2021). However, in Mexico, the health system organization divides the population into two main groups, based on their social security (SS) status. Formal sector workers and their families access SS institutions depending on their workplace, which are the Social Security Mexican Institute (*Instituto Mexicano del Seguro Social, IMSS*), the Institute of Security and Social Services for State Workers (*Instituto de Seguridad y Servicios Sociales de los Trabajadores del Estado, ISSSTE*), the Mexican oil company (*Petróleos Mexicanos, Pemex*), the Secretariat of National Defense (*Secretaría de la Defensa Nacional, SEDENA*), and the Secretariat of the Navy (*Secretaría de Marina, SEMAR*). The population not covered by SS receives care through institutions that often emerge or are restructured depending on the political context. Currently, these include *IMSS-Bienestar* and the private health services. The first is the most recent health policy reform, while the latter serves a smaller share of the population for hospitalizations but covers over half for outpatient care (García-Hernández *et al.* 2024, Gómez-Dantés *et al.* 2024). The main difference between IMSS and *IMSS-Bienestar* is that IMSS belongs to SS and is exclusive to formal workers, while *IMSS-Bienestar* was designed to provide health services to persons outside SS without considering IMSS infrastructure and resources (Santacruz-Varela *et al.* 2024).

The fragmented structure of the Mexican health system results in unequal levels of public health expenditure. Total and per capita public spending have historically been higher for the population covered by SS (Méndez-Méndez 2024). Similar disparities are also evident in the distribution of healthcare resources, including the availability of physicians, nurses, and hospital beds (Centro de Investigación Económica y Presupuestaria 2018, González-Block *et al.* 2021), as well as in differential access to health services (Méndez-Méndez 2022). For example, differences have been reported in the diagnosis and treatment of depressive symptoms (Cerecero-García *et al.* 2020), breast cancer screening (Agudelo Botero 2013, López-Carrillo *et al.* 2014), and the availability of resources for treating chronic kidney disease (Valdez-Ortiz *et al.* 2018).

Access to health systems is essential for older adults, as it can promote healthy aging (McMaughan *et al.* 2020, Cabañero-Garcia *et al.* 2025). Moreover, this age group requires continuous and equitable access to healthcare services due to the complex and costly epidemiological landscape they face. Therefore, the World Health Organization (WHO) has identified integrated healthcare as one of the four strategic areas of action in the Decade of Healthy Ageing 2021–30, aimed at promoting healthy aging globally (Organización Mundial de la Salud 2015 ). However, due to the fragmentation of the Mexican health system, individuals often transition between and within SS and non-SS schemes depending on their employment status throughout their lives. These shifts between SS and non-SS coverage have important repercussions, as periods of informal employment may limit access to comprehensive healthcare and other benefits provided by the SS. It is a common challenge within the health system (Guerra *et al.* 2018 ). This instability undermines the continuity of care, delaying treatment, follow-up, and long-term disease management (García-Hernández *et al.* 2023), which ultimately leads to higher healthcare costs.

Given the role of health systems in ensuring financial security to meet the healthcare needs of older adults, the transitions individuals experience between the two schemes, and the disparities between SS and non-SS institutions, this study aims to analyze the relationship between OOPEs and SS status over time among Mexican adults aged 50 and older.

## Methods

We conducted a panel survey analysis using data from the 2012, 2015, 2018, and 2021 waves of the Mexican Health and Aging Study (MHAS). The MHAS is a nationally representative longitudinal survey of Mexican adults aged 50 and older. Additional information is available on its official website, https://www.mhasweb.org. We considered individuals aged 50 and older in 2021 who were first interviewed in 2012 and subsequently followed in 2015, 2018, and 2021, and who reported having incurred health expenditures in MHAS waves.

### Variables analyzed

The dependent variable was total direct OOPE, defined as the sum of expenditures on medicines, dental consultations, outpatient surgeries, medical consultations, and hospital stays for each year. In the MHAS, expenditures are reported annually, except for medicines, for which expenditures are reported for a typical month. To standardize all components on an annual basis, medicine expenditures were multiplied by 12, assuming a consistent monthly expense (Salinas Escudero *et al.* 2019). All monetary values were adjusted for inflation based on Mexico’s National Consumer Price Index as of December 2024 (Instituto Nacional de Estadística y Geografía n.d.) and subsequently converted to US dollars using the exchange rate of USD 1 = MXN 20.51, corresponding to 31 December 2024 (BANXICO n.d.).

The independent variable was SS stability, categorized as stable, unstable with SS, unstable without SS, and no SS. Participants who reported being affiliated with any SS institution in all MHAS waves were classified as stable. Those who reported SS affiliation in at least one wave, but not in all, were categorized as unstable. Then, if they still had SS in 2021, they were defined as unstable with SS. Conversely, unstable individuals who lost SS by the last wave were considered unstable without SS. Finally, those who never reported SS affiliation in any wave were classified as having no SS.

Additionally, we included covariates identified in the literature as associated with OOPE, which we grouped into sociodemographic and health-related variables. Sociodemographic variables included age (50–59, 60–69, 70–80, and 80 and more), sex, years of schooling (0, 1–6, 7–12, 13–18, 19, or more), marital status (classified as married or in a partnership and single, the latter comprising individuals who are single, divorced, separated, or widowed), current employment status (whether the older adult is still working), self-reported economic situation (categorized as good, fair, or poor), and urbanization level (based on whether the locality has more or fewer than 100 000 inhabitants). Health-related variables included multimorbidity, disability, smoking status, and self-rated health. Multimorbidity was constructed by identifying individuals who reported having two or more of the following chronic conditions: diabetes mellitus, hypertension, respiratory disease, cancer, stroke, cardiovascular disease, and arthritis. Disability was defined as having difficulty performing at least one instrumental activity of daily living (IADL) (shopping, preparing food, managing money, or taking medications) and/or at least one basic activity of daily living (BADL) (eating, dressing, getting in or out of bed, bathing, walking, or using the toilet). Smoking status was categorized according to whether individuals currently smoked or not. Lastly, self-rated health was originally measured using a five-category Likert scale (excellent, very good, good, fair, and poor). We recategorized it into three groups: good (including excellent, very good, and good), fair, and poor.

### Statistical analysis

We conducted a descriptive analysis over time using the mean values of total OOPE and its components, stratified by SS stability, for each of the 2012, 2015, 2018, and 2021 waves. To accurately capture changes in the proportion of adults incurring OOPE over time, we calculated the growth rate using the following formula: (final percentage − initial percentage)/initial percentage × 100. The growth rate allows for a clearer representation of increases or decreases in the proportion of adults with OOPE.

Therefore, to examine the effect of SS stability on total OOPE, we focused on the 2021 data. We describe OOPE in relation to selected covariates, followed by three quantile regression models using the median instead of the mean to account for extreme values and potential skewness in the OOPE distribution. The three models also served as a form of sensitivity analysis, estimating alternative specifications that included specific covariates to assess the stability of the coefficients.

The first model included only the SS stability variable. The second added sociodemographic covariates, and the third incorporated both sociodemographic and health-related variables. Models two and three were developed using a forward stepwise approach, retaining variables with a *P* < .1 as significant contributors. Variables selected through this stepwise procedure were then included in multivariable linear regression models with OOPE as the dependent variable, where SS stability was incorporated using the Enter method (Ranganathan *et al.* 2017). This modeling strategy aligns with the primary objective of the study. To ensure the robustness of our results, we used robust standard errors to account for potential heteroscedasticity and provide consistent estimates of confidence intervals. Additionally, we performed the Wald test for each model to validate overall model significance (Alonso-Pérez and Furio-Blasco 2023) and confirm that the included variables jointly contributed to explaining variation in OOPE. A *P* < .05 was considered statistically significant.

Finally, to further explore the relationship between affiliation to a health institution and the place where individuals receive medical attention among older adults with health expenditures, we conducted a descriptive analysis using 2021 data. This allowed us to estimate the percentage of older adults who incurred OOPE despite using the services provided by the institution to which they were affiliated. The results are presented in Supplementary File S1.

All analyses were performed using Stata version 17.

## Results

The 2021 wave included a sample of 9251 adults aged 50 and older. This sample consisted of individuals who were followed across the 2012, 2015, and 2018 waves, which included 8791, 8993, and 9161 observations, respectively. The difference of 460 individuals between the 2012 and 2021 waves corresponds to participants who reached the age of 50 or older during this period and were therefore included in the 2021 analysis. Table 1 presents the mean age for each wave, the percentage of participants who incurred OOPE, and the mean OOPE broken down by components and stratified by SS stability categories.

**Table 1:** Mean OOPE broken down by its components, stratified by SS stability categories.

	2012 (*n* = 8791)		2015 (*n* = 8993)		2018 (*n* = 9161)		2021 (*n* = 9251)	
Mean age	63.33		65.61		68.35		77.23	
	Exp.	SD	Exp.	SD	Exp.	SD	Exp.	SD
Total OOPE^a^	54.01%		60.88%		61.86%		69.54%	
Stable	1309.21	12 807.50	943.98	2524.65	1214.31	4985.09	1405.13	3309.18
Unstable with SS	–	–	1115.70	5396.89	1022.05	2451.90	1375.03	4476.09
Unstable without SS	–	–	782.91	1222.09	1961.85	8864.88	1152.31	3067.76
Without SS	1237.18	6290.59	912.55	3118.03	1343.10	5773.20	1163.16	3024.84
Total	1277.05	10 413.51	936.10	2974.24	1287.53	5421.86	1308.87	3394.33

Medicines^a^	37.80%		43.09%		44.19%		52.11%	
Stable	1557.98	15 830.36	920.75	2501.98	1223.18	5778.23	1222.53	3195.40
Unstable with SS	–	–	1142.13	6108.51	925.01	2438.51	1296.05	4801.27
Unstable without SS	–	–	784.64	1253.37	1952.40	9750.90	918.47	2402.76
Without SS	1344.87	6938.99	873.82	3308.65	1314.60	6314.15	1019.95	2909.24
Total	1452.93	12 278.99	909.94	3149.45	1278.36	6115.13	1143.66	3315.32

Dental consultations^a^	22.82%		25.74%		26.35%		24.82%	
Stable	299.48	1474.29	258.73	485.63	306.90	592.85	373.36	948.61
Unstable with SS	–	–	219.02	283.74	248.10	508.88	257.35	700.71
Unstable without SS	–	–	203.56	346.46	241.22	635.76	191.33	334.39
Without SS	189.49	570.71	177.22	318.50	224.93	847.88	226.58	537.77
Total	263.84	1255.78	232.10	432.28	278.18	657.14	313.32	814.81

Outpatient surgeries^a^	1.23%		1.71%		2.26%		2.25%	
Stable	958.12	1356.84	950.09	1403.62	1012.65	1634.15	1249.22	1086.54
Unstable with SS	–	–	749.59	844.07	907.42	1188.95	1138.66	1148.99
Unstable without SS	–	–	621.89	797.89	1143.07	1037.17	865.31	1288.33
Without SS	924.12	1398.33	614.58	902.04	759.43	1120.02	935.04	1304.89
Total	945.84	1365.53	783.12	1160.43	935.59	1431.61	1133.36	1166.37

Medical consultations^a^	21.98%		25.90%		25.13%		36.57%	
Stable	218.93	833.61	179.96	418.30	258.98	686.95	308.39	795.56
Unstable with SS	–	–	160.11	365.95	257.98	868.32	250.29	591.07
Unstable without SS	–	–	117.76	193.10	252.64	807.58	160.07	428.08
Without SS	128.17	342.51	130.92	284.11	226.79	837.47	203.22	428.75
Total	169.91	620.30	154.29	353.56	245.40	776.29	251.25	634.45

Hospitalizations^a^	2.02%		3.08%		3.56%		4.11%	
Stable	2251.00	3334.99	2758.36	3642.08	2726.21	3701.23	3035.26	4020.85
Unstable with SS	–	–	2349.06	5619.58	1995.18	2468.47	3301.35	3897.03
Unstable without SS	–	–	886.58	718.89	2378.79	3649.18	3797.69	5032.79
Without SS	1133.17	1323.09	1622.80	2891.15	1616.31	2503.76	2356.89	2308.07
Total	1547.65	2339.95	2077.66	3383.64	2121.27	3128.68	2898.07	3626.69

Exp, expenditure in USD; SD, standard deviation in USD; SS, social security.

^a^Each year shows the percentage of the yearly sample of 50 years or more (total sample of 8791 in 2012, 8993 in 2015, 9161 in 2018, and 9251 in 2021) that incurred expenses in total OOPE, medicines, dental consultations, outpatient surgeries, medical consultations, and hospitalizations.

The average age increased over time. Also, the percentage of older adults who reported OOPE increased steadily across all categories. The largest increase, represented as the growth rate, was observed in hospitalization expenditures (103.47%), outpatient surgeries (82.93%), medical consultations (66.38%), medicine (37.86%), and dental consultations (8.76%). The mean amounts spent on total OOPE and its components rose between 2012 and 2021.

When stratified by SS stability, individuals without SS reported the lowest mean expenditures across all components. In contrast, individuals with stable SS showed a steady increase in spending throughout the period, ultimately reporting the highest mean expenditures on total OOPE, dental consultations, outpatient surgeries, and medical consultations by 2021. However, older adults classified as unstable without SS experienced the largest increase in hospital expenditures over time.

Table 2 presents a descriptive analysis of the mean OOPE for 2021 with covariates, among individuals who reported any expenditure. The results show that total OOPE is higher in adults with SS stability. Also, it increases with age, and men tend to spend more than women. Similarly, individuals with higher educational attainment and a better self-reported economic situation tend to exhibit higher expenditures. Those married, not currently working, and living in more urbanized areas also report greater health spending. Regarding health-related variables, OOPE increases with the presence of chronic conditions, disability, and poor self-rated health status.

**Table 2:** Descriptive analysis of the mean OOPE for 2021 with the covariates considered, among individuals who reported any expenditure.

	Total OOPE (mean)	6433 observations in 2021 (%)
SS stability		
Stable	1405.13	48.75
Unstable with SS	1375.03	13.48
Unstable without SS	1152.31	7.38
Without SS	1163.16	30.39
Age		
50–59	1049.84	9.41
60–69	1142.32	35.67
70–79	1437.80	36.56
80 and more	1506.34	18.36
Sex		
Men	1372.35	37.51
Women	1270.77	62.49
Years of school		
0	1122.71	16.06
1–6	1280.67	51.78
7–12	1290.04	22.48
13–18	1802.34	9.01
19 and more	1943.02	0.67
Marital status		
Married or in union	1345.82	58.84
Single	1256.05	41.16
Employment status		
Working	994.01	25.88
Not working	1418.82	74.12
Economic situation^a^		
Good	1409.83	31.97
Fair	1114.80	61.41
Poor	1120.49	6.62
Locality size		
>100 000 inhabitants	1402.67	54.00
<100 000 inhabitants	1198.74	46.00
Morbidity		
0	1002.15	28.01
1	1240.41	37.40
2	1631.31	34.59
Disability		
No	1114.41	66.78
Yes	1701.52	33.22
Smoking status		
No	1334.10	91.71
Yes	1029.63	8.29
Self-rated health status^b^		
Good	1056.35	31.61
Fair	1189.09	57.91
Poor	1793.46	10.48

SS, social security.

^a^The sample is 5786 observations because not all participants answered this question.

^b^The sample is 5802 observations because not all participants answered this question.

Table 3 presents the results of the three quantile regression models. The SS stability variable was statistically significant in the first model, where being without SS or being unstable to SS coverage was associated with a median reduction of 94.66 USD in OOPE. In Model 2, each additional year of age and education was associated with an increase of 5.15 USD and 12.50 USD, respectively, in the median OOPE. Individuals who were not working reported a 119.64 USD higher median OOPE than those who were working, and persons with a fair economic situation spent a median of 53.44 USD less than those with a good situation. In Model 3, SS stability, age, years of education, working status, and economic situation remained significantly associated with OOPE. However, the health-related variables showed higher values. Older adults with multimorbidity had a 189.11 USD higher median OOPE compared to those without chronic conditions. Similarly, the presence of disability was associated with an increase of 110.87 USD, and those with poor self-rated health status spent 275.62 USD more in median terms. The Wald test for all models was statistically significant, indicating that the included variables have a valid influence in determining OOPE and are important predictors of this outcome. Therefore, the models are appropriate and accurately represent the relationship between the independent variables and OOPE.

**Table 3:** Quantile regression models for OOPE in 2021.

	Model 1 (*n* = 6433)	Model 2 (*n* = 5768)	Model 3 (*n* = 5765)
	OR CI 95%	OR CI 95%	OR CI 95%
SS stability			
Stable	Reference		
Unstable with SS	−94.66** (−149.37 to −39.96)	−30.97 (−77.45 to 15.51)	−35.35 (−71.59 to 0.90)
Unstable without SS	−94.66** (−150.77 to −38.56)	−57.65 (−122.37 to 7.06)	−60.67* (−118.99 to 2.39)
Without SS	−94.66** (−137.24 to −52.09)	−40.50* (−79.28 to −1.71)	−45.33* (−79.06 to 11.62)
Age^a^		5.15** (3.18–7.13)	3.24** (1.47–5.02)
Sex			
Men		Reference	
Women		—	—
Years of school^a^		12.40** (7.85–16.95)	16.39** (12.36–20.42)
Marital status			
Married or in union		Reference	
Single		–	–
Employment status			
Working		Reference	
Not working		119.64** (87.89–151.38)	67.41** (38.79–96.03)
Economic situation			
Good		Reference	
Fair		−53.44* (−99.27 to −7.60)	−87.19** (−132.17 to −42.21)
Poor		−45.77 (−97.70 to 6.14)	−94.81** (−147.61 to −42.01)
Locality size			
>100 000 inhabitants		Reference	
<100 000 inhabitants		–	–
Morbidity			
None			Reference
1			53.55** (26.39–80.70)
2			189.11** (141.10–237.11)
Disability			
No			Reference
Yes			110.87** (68.43–153.32)
Smoking status			
No			Reference
Yes			—
Self-rated health status			
Good			Reference
Fair			70.20** (43.19–97.21)
Poor			275.62** (171.33–379.90)
Wald test (*P* value)	0.0001	0.0000	0.0000

SS, social security.

^a^These variables were included as continuous in the regression models.

^*^ *P* < .05; **P < .01.

Supplementary File S1 presents the relationship between health system affiliation and the place where individuals received medical attention among older adults with health expenditures in 2021. A considerable percentage of people engaged with SS institutions (IMSS, ISSSTE, PEMEX/SEDENA/SEMAR) have OOPE, specifically 72.34% of dental consultations, 67.79% of outpatient surgeries, 58.16% of hospitalizations, and 56.16% of medical consultations. The data also show a substantial proportion of care being provided by the private sector. Additionally, the presence of pharmacy-attached clinics and the use of alternative providers, such as homeopathic practitioners, traditional healers, and chiropractors, highlight additional sources of OOPE for medical consultations. Medicines were excluded from this analysis, as the MHAS does not include a specific question that allows us to determine whether medications were obtained through a public health institution or purchased independently.

## Discussion

Financial protection is one of the essential functions of health systems (Murray and Frenk 2000), as it helps to prevent excessive health spending that may lead to impoverishment. This issue is particularly relevant for older adults, given their greater healthcare needs. However, the final aim of improving and/or keeping the population health is far from being reached in every society. Mexico is not the exception. Our findings indicate that older adults with stable SS coverage reported the highest OOPE in 2021, including total expenditures, dental consultations, outpatient surgeries, and medical consultations. Additionally, the first and third regression models revealed that those with SS stability incurred significantly higher OOPE compared to other groups.

This finding contrasts with international evidence on the protective role of health insurance. Numerous studies have shown that insurance programs can significantly reduce OOPE among older adults. For example, in the USA, Medicaid, a public insurance program targeting vulnerable populations, was associated with a reduction of 528 USD in total health spending (Fong 2019). In Vietnam, insured individuals experienced a 32% reduction in OOPE compared to their uninsured counterparts (Thanh *et al.* 2019). Similarly, in Colombia, households without insurance face a greater risk of incurring catastrophic health expenditures (Amaya-Lara 2016), while in Ghana, health insurance coverage reduced the likelihood of households falling into poverty due to health spending by 7.5% (Aryeetey *et al.* 2016). Also, in China, insurance coverage for vulnerable older adults has been linked to lower medical expenses (Zeng *et al.* 2019).

An analysis of OOPE in Mexico, using data from the National Health and Nutrition Survey (ENSANUT) 2018 and 2021, found that Mexicans experienced an increase in mean total health expenditures. This increase was >90% among individuals affiliated with SS institutions, compared with a 60% increase among those without SS coverage (Knaul *et al.* 2023). Our results are consistent with these findings, showing that people affiliated with SS institutions reported an increase in OOPE. However, the reasons for this rise are multifactorial and likely reflect the interaction of several elements. We hypothesize four possible explanations for this trend.

First, health expenditure has progressively increased among households headed by older adults (México Evalúa 2025). This may be associated with the epidemiological profile of this population, characterized by a high prevalence of chronic diseases and disability, which implies a growing demand for healthcare services (Parra-Rodríguez *et al.* 2020). Recent studies suggest that individuals affiliated with SS institutions may present poorer health status, reflected in higher rates of mortality (Ventura-Alfaro *et al.* 2016, Ascencio-Montiel 2020, Aldaco-Sarvide *et al.* 2022, García-Hernández and Dávila-Cervantes 2022, García-Hernández *et al.* 2023) and disability (Cabrero Castro *et al.* 2021). Second, the legislative and structural changes in the Mexican health system following the 2018 political transition were aimed at strengthening health services for people without SS coverage, while SS institutions largely retained their existing structure. For instance, the General Health Law was modified to guarantee free provision of healthcare services, medicines, and associated inputs for individuals without SS. Additionally, there was the elimination of *Seguro Popular (SP)*, a program that provided limited coverage under the *Catálogo Universal de Servicios de Salud (CAUSES)*, and the creation of the *Instituto de Salud para el Bienestar (INSABI)* sought to expand the range of services offered and provide care for all diseases while increasing the health resources for this new institution (Reich 2020, García-Hernández and Esquer-Bojorquez 2024, Santacruz-Varela *et al.* 2024). Evidence also suggests that SP may not have significantly reduced health expenditures among the uninsured population (Sovilla and Díaz-Sánchez 2022). Indeed, during SP implementation, the use of outpatient services by noninsured individuals at pharmacy-based medical offices increased (Bautista-Arredondo *et al.* 2024). It should be noted that these structural changes are recent, and their impact on the health of the uninsured population, and on OOPE, requires further evaluation. Third is a reduction of access to public healthcare services during the COVID-19 (coronavirus disease) pandemic. This trend, also observed internationally in countries such as Singapore (Malhotra *et al.* 2021) and Germany (Michalowsky *et al.* 2021), was evident in Mexico as well (Colchero *et al.* 2021, García-Hernández *et al.* 2024). During the pandemic, public health institutions prioritized the care of COVID-19 patients, prompting a shift toward private providers. For example, the proportion of IMSS-affiliated individuals using private services increased from 31% in 2012 to 39% in 2021, while for ISSSTE affiliates, it rose from 37% in 2018 to 58% in 2021. The use of private healthcare services is directly associated with higher health expenditures (Knaul *et al.* 2023). Fourth, the longstanding oversaturation and limited availability of health resources within the public health system leads people to prefer private services for acute health problems, often through pharmacy-based medical offices (Colchero *et al.* 2021). This reflects the system’s insufficient capacity to meet population health needs. For instance, Mexico reports lower ratios of hospital beds, physicians, nurses, and high-technology medical equipment per 1000 inhabitants compared to both the Organisation for Economic Co-operation and Development (OECD) and Latin American regional benchmarks. Moreover, healthcare personnel are unevenly distributed, with high concentrations in certain states of the country (Gómez-Dantés *et al.* 2024).

On the other hand, the increase in health expenditures among noninsured individuals could also be partially explained by the implementation of nonconditional cash transfer programs, such as the universal pension for older adults. During the 2018–24 political administration, Article 4 of the Mexican Constitution was modified to guarantee these transfers, primarily benefiting people outside the formal economy who lack access to contributory pensions in old age (Rodríguez-Gómez 2024). Previous evidence has shown that pension income can increase the use of formal healthcare services and reduce financial barriers to accessing primary care (Riumallo-Herl and Aguila 2019). Therefore, these programs may have significantly benefited noninsured older adults by enabling greater healthcare utilization.

Regarding the expenditures of every OOPE component, medicines were the main cost driver, which is consistent with existing evidence (Sesma-Vázquez *et al.* 2005, Brinda *et al.* 2015, Nikoloski and Mossialos 2018, Fong 2019, Larkin *et al.* 2025). In Mexico, previous studies have found that medicines account for ∼70% of OOPE (Sesma-Vázquez *et al.* 2005, Salinas Escudero *et al.* 2019). Our findings indicate that having SS does not necessarily reduce this cost. Moreover, although hospitalization was a least frequent category of healthcare use, it represented the largest single expenditure for older Mexicans regardless of their SS status. As shown in Supplementary Material S1, this cost is primarily associated with the use of private healthcare services, which have been linked not only to higher OOPE but also to catastrophic health expenditures (Sesma-Vázquez *et al.* 2005, Brinda *et al.* 2015, Amaya-Lara 2016). For example, in India, average OOPE in the private sector was found to be six times higher than in the public sector (Garg *et al.* 2022). These findings may reflect persistent barriers to accessing care within Mexico’s public healthcare system, including structural challenges such as long waiting times, overcrowding, poor service quality, and limited availability of medical equipment (Salinas Escudero *et al.* 2019, García-Hernández *et al.* 2024).

Concerning covariates, we found that each additional year of age is associated with an increase in OOPE. However, the evidence on this relationship is mixed. Different studies, treating age either as a categorical or continuous variable, have reported inconsistent results. For example, no significant relationship was found in studies conducted in India (Brinda *et al.* 2015), Tanzania (Brinda *et al.* 2014), China (Zeng *et al.* 2019), the USA (Fong 2019), and Denmark (Larkin *et al.* 2025). In contrast, other studies, such as those in Colombia (Amaya-Lara 2016) or Vietnam (Thanh *et al.* 2019), reported a positive association between age and OOPE. These discrepancies may reflect variations in healthcare systems, the existence of targeted programs for older adults, or the strength of welfare states in reducing financial barriers to healthcare. Countries with robust welfare systems, such as those in Europe, often aim to provide a social safety net and support for individuals in disadvantaged situations (Sieber *et al.* 2022). So higher levels of social protection in these contexts can help reduce health spending for the elderly.

Another variable with inconsistent findings regarding its relationship with OOPE was sex. Internationally, being a woman is often associated with higher OOPE (Fong 2019, Thanh *et al.* 2019, Zeng *et al.* 2019, Larkin *et al.* 2025). This association may be partly explained by the longer life expectancy of older women, which increases their likelihood of experiencing multiple health problems and, therefore, needing more healthcare services (Chirinda and Chen 2017, Moreno *et al.* 2020, Wang *et al.* 2021). However, other studies, including ours, find no significant association between female sex and increased OOPE (Brinda *et al.* 2014, 2015). A more in-depth analysis is required using a sophisticated framework such as intersectionality. This approach could help unpack how health outcomes and healthcare expenditures are shaped not only by biological sex but also by the social meanings and roles associated with being a woman in contemporary societies (García-Hernández *et al.* 2025).

On the other hand, there is a consensus regarding the relationship between education, income, and work with OOPE. Multiple studies have shown that OOPE tends to increase among individuals with higher income, higher education, and those who are not currently employed (Brinda *et al.* 2014, Amaya-Lara 2016, Fong 2019, Thanh *et al.* 2019, Zeng *et al.* 2019, Larkin *et al.* 2025). For example, in the USA, older adults in the top income quartile spend an average of 374.8 USD more on healthcare than those in the bottom quartile (Fong 2019). Similarly, in India, individuals aged 65 or older without formal education spend on average 15.5 USD less than those with formal education (Brinda *et al.* 2015). In Colombia, older adults who work are 66% less likely to incur catastrophic health expenditures than their nonworking counterparts (Amaya-Lara 2016). The association between income, education, and employment with health spending may be explained by greater health awareness, increased healthcare-seeking behavior, greater economic resources, and improved access to private services. Consequently, socioeconomic disadvantages can further deteriorate an individual’s health status, reinforcing a vicious cycle in which access to healthcare becomes increasingly limited as health needs grow more complex, requiring higher expenses and greater health literacy.

Also, the relationship between morbidity, disability, and increased OOPE is well established and widely analyzed in the scientific literature (Brinda *et al.* 2014, 2015, Islam *et al.* 2017, Fong 2019, Larkin *et al.* 2025). Our research shows that individuals with multimorbidity and disability spend ∼600 USD more than those in good health. Although less frequently studied, self-rated health status is also significantly associated with OOPE. In the USA, older adults who report poor self-rated health spend an average of 227.1 USD more than those who rate their health as excellent (Fong 2019); similar findings were observed in China (Zeng *et al.* 2019).

Given the strong relationship between disease burden and OOPE, the statistical significance of the SS stability in our Model 3 is relevant. This may advise that individuals with SS coverage tend to have a better economic situation. Previous research has shown that this group has higher educational levels (Pérez *et al.* 2025), and the association between education and income has been well documented in the literature (Mou 2023). Therefore, this population is more able to allocate additional resources to healthcare. It could also reflect a tendency among older adults with SS coverage to seek private healthcare services, possibly due to structural deficiencies in the public sector, a pattern that has also been documented in countries such as India (Brinda *et al.* 2015). However, these results may suggest that SS in Mexico does not provide adequate financial protection. In fact, it appears to fall short of fulfilling its role in restoring and maintaining population health, as previously reported (Cabrero Castro *et al.* 2021, García-Hernández and Dávila-Cervantes 2022, García-Hernández *et al.* 2023, 2025). Therefore, it is essential to reassess the current role of the Mexican health system from two perspectives. First, increase the focus on preventive rather than curative care, as the latter tends to be more costly. Implementing this change of perspective about the health system may help reduce OOPE in the medium and long term. Second, conduct a deeper analysis of waiting times and healthcare quality in SS institutions to better understand and address the factors that may lead individuals with SS to prefer receiving care in private facilities.

To our knowledge, this is the first study to analyze the continuity of SS over time in relation to out-of-pocket expenditures. However, several limitations must be acknowledged. First, our analysis focuses solely on direct OOPE and does not include indirect costs, such as transportation expenses, income lost due to missed workdays, or the reduction in productivity and earnings associated with illness. Second, the breakdown of direct OOPE components is limited, excluding important categories such as laboratory and imaging tests, emergency services, rehabilitation, and preventive care, all of which may significantly impact total OOPE. Third, we were unable to estimate catastrophic health expenditures, as the MHAS does not include questions about the proportion of income allocated to health care. Fourth, there is a potential for recall bias, as both OOPE and health conditions are self-reported and not clinically validated. Fifth, we considered SS affiliation; however, this variable may not accurately reflect individuals’ actual access to the health services to which they are entitled. Finally, MHAS does not collect information on the source from which individuals received their medications, limiting further analysis in this area (see Supplementary Material S1).

## Conclusion

SS affiliation was not associated with lower OOPE. In fact, older adults with stable SS coverage spent more than those with unstable or no SS coverage. The explanation for these findings is multifactorial. It may be related to the greater demand for healthcare services among older adults with SS, due to their likely poorer epidemiological profile characterized by chronic diseases and disabilities. Additionally, structural changes in the Mexican health system in 2018 that were focused on strengthening services for people without SS may have affected access and utilization patterns. Reduced availability of public healthcare services during the COVID-19 pandemic, along with the longstanding overcrowding and limited resources in public institutions, may also have led individuals to seek private care for acute conditions. Medicines represented the largest component of OOPE; however, despite being a least frequent type of healthcare use, hospitalizations were the highest single expense for older Mexicans. This may indirectly indicate persistent barriers to accessing public healthcare and underscore the central role of the private sector in healthcare provision. Further research is needed to assess the impact of OOPE on impoverishment.

## Supplementary Material

czaf103_Supplementary_Data

## Data Availability

Data from the Mexican Health and Aging Study (MHAS) is available on its official website: https://www.mhasweb.org.

## Reflexivity statement

This work was carried out by a team composed of two senior researchers (a woman and a man) and one early-career researcher. The senior authors acted as mentors, providing guidance in shaping and advancing the research idea. Their mentorship supported the younger author in articulating his ideas and gaining recognition for his contribution. Furthermore, this project involved two research institutions, strengthening the interdisciplinary nature of the study. All authors focus their research on a vulnerable group—older adults—who are often overlooked in the public agenda of middle-income countries such as Mexico.
