# Supplementary material for: Out-of-pocket healthcare expenditures in older Mexican people based on their social security status
Source: Health Policy Plan. 2025 Dec 3;41(2):252–61. doi: 10.1093/heapol/czaf103 (PMC12906755; doi:10.1093/heapol/czaf103)
Supplement: czaf103_Supplementary_Data [file czaf103_supplementary_data.zip › Suppl1.docx]

Supplementary File 1. Relation between health system affiliation and the place where individuals received medical attention among older adults with health expenditures in 2021.

|  |  | Place where the individuals received health service | | | | | | | | |
| --- | --- | --- | --- | --- | --- | --- | --- | --- | --- | --- |
| Affiliation to health institution | Observations  n (%) | IMSS  (%) | ISSSTE  (%) | SSA  (%) | IMSS-B  (%) | PEMEX  SEDENA  SEMAR  (%) | Private  (%) | Pharmacy-attached clinics  (%) | Homeopathic practitioners  Traditional healers  Chiropractor  (%) | Other  (%) |
| Dental consultations | 2,296 |  | | | | | | | | |
| IMSS | 1,245 (54.22) | 0.16 | 0.08 | 0.48 | 0.00 | 0.00 | 92.29 | 1.93 | 0.00 | 5.06 |
| ISSSTE | 380 (16.55) | 0.00 | 1.32 | 0.26 | 0.00 | 0.00 | 93.95 | 1.58 | 0.00 | 2.89 |
| PEMEX SEDENA SEMAR | 36 (1.57) | 0.00 | 0.00 | 2.78 | 0.00 | 2.77 | 88.89 | 2.78 | 0.00 | 2.78 |
| SSA | 175 (7.62) | 0.00 | 0.00 | 4.00 | 0.58 | 0.00 | 89.14 | 5.14 | 0.00 | 1.14 |
| Private | 14 (0.61) | 0.00 | 0.00 | 0.00 | 0.00 | 0.00 | 100.00 | 0.00 | 0.00 | 0.00 |
| Other | 19 (0.83) | 0.00 | 0.00 | 0.00 | 0.00 | 0.00 | 100.00 | 0.00 | 0.00 | 0.00 |
| Without | 427 (18.60) | 0.23 | 0.00 | 1.64 | 0.23 | 0.00 | 89.93 | 2.81 | 0.00 | 5.16 |
|  | | | | | | | | | | |
| Outpatient surgeries | 208 |  | | | | | | | | |
| IMSS | 114 (54.81) | 1.75 | 0.88 | 2.63 | 0.00 | 0.00 | 92.98 | 0.88 | 0.00 | 0.88 |
| ISSSTE | 23 (11.06) | 0.00 | 13.04 | 0.00 | 0.00 | 0.00 | 82.61 | 0.00 | 0.00 | 4.35 |
| PEMEX SEDENA SEMAR | 4 (1.92) | 0.00 | 0.00 | 0.00 | 0.00 | 0.00 | 100.00 | 0.00 | 0.00 | 0.00 |
| SSA | 17 (8.17) | 5.88 | 0.00 | 0.00 | 5.88 | 0.00 | 64.71 | 17.65 | 0.00 | 5.88 |
| Private | 4 (1.92) | 0.00 | 0.00 | 0.00 | 0.00 | 0.00 | 100.00 | 0.00 | 0.00 | 0.00 |
| Other | 1 (0.48) | 0.00 | 0.00 | 0.00 | 0.00 | 0.00 | 0.00 | 100.00 | 0.00 | 0.00 |
| Without | 45 (21.63) | 0.00 | 0.00 | 2.22 | 0.00 | 0.00 | 97.78 | 0.00 | 0.00 | 0.00 |
|  | | | | | | | | | | |
| Medical consultations | 3,383 |  | | | | | | | | |
| IMSS | 1,399 (41.35) | 2.29 | 0.00 | 0.21 | 0.00 | 0.07 | 73.70 | 19.23 | 1.07 | 3.43 |
| ISSSTE | 453 (13.39) | 0.00 | 2.21 | 0.44 | 0.00 | 0.00 | 76.60 | 17.00 | 0.88 | 2.87 |
| PEMEX SEDENA SEMAR | 48 (1.42) | 0.00 | 0.00 | 0.00 | 0.00 | 0.00 | 68.75 | 22.92 | 2.08 | 6.25 |
| SSA | 357 (10.55) | 0.00 | 0.00 | 5.32 | 1.40 | 0.00 | 64.43 | 25.21 | 0.00 | 3.64 |
| Private | 16 (0.47) | 0.00 | 0.00 | 0.00 | 0.00 | 0.00 | 93.75 | 6.25 | 0.00 | 0.00 |
| Other | 19 (0.56) | 0.00 | 0.00 | 10.53 | 0.00 | 0.00 | 57.89 | 10.53 | 0.00 | 21.05 |
| Without | 1,091 (32.25) | 0.09 | 0.00 | 3.12 | 0.09 | 0.00 | 68.29 | 22.64 | 1.19 | 4.58 |
|  | | | | | | | | | | |
| Hospitalizations | 380 |  | | | | | | | | |
| IMSS | 175 (46.05) | 18.29 | 0.57 | 4.00 | 0.00 | 0.00 | 74.85 | 0.00 | 0.00 | 2.29 |
| ISSSTE | 45 (11.84) | 0.00 | 15.56 | 8.89 | 0.00 | 0.00 | 73.33 | 0.00 | 0.00 | 2.22 |
| PEMEX SEDENA SEMAR | 1 (0.26) | 0.00 | 0.00 | 0.00 | 0.00 | 0.00 | 100.00 | 0.00 | 0.00 | 0.00 |
| SSA | 38 (10.00) | 5.26 | 0.00 | 7.89 | 5.26 | 0.00 | 76.33 | 0.00 | 0.00 | 5.26 |
| Private | 4 (1.05) | 0.00 | 0.00 | 0.00 | 0.00 | 0.00 | 100.00 | 0.00 | 0.00 | 0.00 |
| Other | 2 (0.53) | 0.00 | 0.00 | 0.00 | 0.00 | 0.00 | 50.00 | 0.00 | 0.00 | 50.00 |
| Without | 115 (30.26) | 1.74 | 0.00 | 14.78 | 1.74 | 0.00 | 79.13 | 0.00 | 0.00 | 2.61 |

*Medicines were not included because the MHAS does not contain a specific question that allows us to determine whether individuals obtained their medications from a health institution or purchased them independently. In contrast, MHAS does include questions regarding components such as dental consultations, outpatient surgeries, medical consultations, and hospitalizations, which specify the place where the individual received care.

IMSS- Social Security Mexican Institute (*Instituto Mexicano del Seguro Social)*.

ISSSTE- Institute of Security and Social Services for State Workers *(Instituto de Seguridad y Servicios Sociales de los Trabajadores del Estado).*

PEMEX- Mexican oil company *(Petróleos Mexicanos)*.

SEDENA- Secretariat of National Defense *(Secretaría de la Defensa Nacional)*.

SEMAR- Secretariat of the Navy *(Secretaría de Marina)*.

SSA- Ministry of Health *(Secretaría de Salud)*.
