# Supplementary material for: Out-of-pocket healthcare expenditures in older Mexican people based on their social security status
Source: Health Policy Plan. 2025 Dec 3;41(2):252–61. doi: 10.1093/heapol/czaf103 (PMC12906755; doi:10.1093/heapol/czaf103)
Supplement: czaf103_Supplementary_Data [file czaf103_supplementary_data.zip › Table 1.docx]

Table 1. Mean OOPE broken down by its components, stratified by SS stability categories.

|  | 2012  (n= 8,791) | | 2015  (n= 8,993) | | 2018  (n= 9,161) | | 2021  (n= 9,251) | |
| --- | --- | --- | --- | --- | --- | --- | --- | --- |
| Mean Age | 63.33 | | 65.61 | | 68.35 | | 77.23 | |
|  | Exp. | SD | Exp. | SD | Exp. | SD | Exp. | SD |
| Total OOPE* | 54.01% | | 60.88% | | 61.86% | | 69.54% | |
| Stable | 1,309.21 | 12,807.50 | 943.98 | 2,524.65 | 1,214.31 | 4,985.09 | 1,405.13 | 3,309.18 |
| Unstable with SS | - | - | 1,115.70 | 5,396.89 | 1,022.05 | 2,451.90 | 1,375.03 | 4,476.09 |
| Unstable without SS | - | - | 782.91 | 1,222.09 | 1,961.85 | 8,864.88 | 1,152.31 | 3,067.76 |
| Without SS | 1,237.18 | 6,290.59 | 912.55 | 3,118.03 | 1,343.10 | 5,773.20 | 1,163.16 | 3,024.84 |
| Total | 1,277.05 | 10,413.51 | 936.10 | 2,974.24 | 1,287.53 | 5,421.86 | 1,308.87 | 3,394.33 |
|  | | | | | | | | |
| Medicines* | 37.80% | | 43.09% | | 44.19% | | 52.11% | |
| Stable | 1,557.98 | 15,830.36 | 920.75 | 2,501.98 | 1,223.18 | 5,778.23 | 1,222.53 | 3,195.40 |
| Unstable with SS | - | - | 1,142.13 | 6,108.51 | 925.01 | 2,438.51 | 1,296.05 | 4,801.27 |
| Unstable without SS | - | - | 784.64 | 1,253.37 | 1,952.40 | 9,750.90 | 918.47 | 2,402.76 |
| Without SS | 1,344.87 | 6,938.99 | 873.82 | 3,308.65 | 1,314.60 | 6,314.15 | 1,019.95 | 2,909.24 |
| Total | 1,452.93 | 12,278.99 | 909.94 | 3,149.45 | 1,278.36 | 6,115.13 | 1,143.66 | 3,315.32 |
|  | | | | | | | | |
| Dental consultations* | 22.82% | | 25.74% | | 26.35% | | 24.82% | |
| Stable | 299.48 | 1474.29 | 258.73 | 485.63 | 306.90 | 592.85 | 373.36 | 948.61 |
| Unstable with SS | - | - | 219.02 | 283.74 | 248.10 | 508.88 | 257.35 | 700.71 |
| Unstable without SS | - | - | 203.56 | 346.46 | 241.22 | 635.76 | 191.33 | 334.39 |
| Without SS | 189.49 | 570.71 | 177.22 | 318.50 | 224.93 | 847.88 | 226.58 | 537.77 |
| Total | 263.84 | 1255.78 | 232.10 | 432.28 | 278.18 | 657.14 | 313.32 | 814.81 |
|  | | | | | | | | |
| Outpatient surgeries* | 1.23% | | 1.71% | | 2.26% | | 2.25% | |
| Stable | 958.12 | 1,356.84 | 950.09 | 1,403.62 | 1,012.65 | 1,634.15 | 1,249.22 | 1,086.54 |
| Unstable with SS | - | - | 749.59 | 844.07 | 907.42 | 1,188.95 | 1,138.66 | 1,148.99 |
| Unstable without SS | - | - | 621.89 | 797.89 | 1,143.07 | 1,037.17 | 865.31 | 1,288.33 |
| Without SS | 924.12 | 1,398.33 | 614.58 | 902.04 | 759.43 | 1,120.02 | 935.04 | 1,304.89 |
| Total | 945.84 | 1,365.53 | 783.12 | 1,160.43 | 935.59 | 1,431.61 | 1,133.36 | 1,166.37 |
|  | | | | | | | | |
| Medical consultations* | 21.98% | | 25.90% | | 25.13% | | 36.57% | |
| Stable | 218.93 | 833.61 | 179.96 | 418.30 | 258.98 | 686.95 | 308.39 | 795.56 |
| Unstable with SS | - | - | 160.11 | 365.95 | 257.98 | 868.32 | 250.29 | 591.07 |
| Unstable without SS | - | - | 117.76 | 193.10 | 252.64 | 807.58 | 160.07 | 428.08 |
| Without SS | 128.17 | 342.51 | 130.92 | 284.11 | 226.79 | 837.47 | 203.22 | 428.75 |
| Total | 169.91 | 620.30 | 154.29 | 353.56 | 245.40 | 776.29 | 251.25 | 634.45 |
|  | | | | | | | | |
| Hospitalizations* | 2.02% | | 3.08% | | 3.56% | | 4.11% | |
| Stable | 2,251.00 | 3,334.99 | 2,758.36 | 3,642.08 | 2,726.21 | 3,701.23 | 3,035.26 | 4,020.85 |
| Unstable with SS | - | - | 2,349.06 | 5,619.58 | 1,995.18 | 2,468.47 | 3,301.35 | 3,897.03 |
| Unstable without SS | - | - | 886.58 | 718.89 | 2,378.79 | 3,649.18 | 3,797.69 | 5,032.79 |
| Without SS | 1,133.17 | 1,323.09 | 1,622.80 | 2,891.15 | 1,616.31 | 2,503.76 | 2,356.89 | 2,308.07 |
| Total | 1,547.65 | 2,339.95 | 2,077.66 | 3,383.64 | 2,121.27 | 3,128.68 | 2,898.07 | 3,626.69 |

Exp- Expenditure in USD.

SD- Standard deviation in USD.

SS- Social security.

*Each year shows the percentage of the yearly sample of 50 years or more (total sample of 8,791 in 2012, 8,993 in 2015, 9,161 in 2018, and 9,251 in 2021) that incurred expenses in Total OOPE, Medicines, Dental Consultations, Outpatient surgeries, Medical consultations, and Hospitalizations.
