# Supplementary material for: Out-of-pocket healthcare expenditures in older Mexican people based on their social security status
Source: Health Policy Plan. 2025 Dec 3;41(2):252–61. doi: 10.1093/heapol/czaf103 (PMC12906755; doi:10.1093/heapol/czaf103)
Supplement: czaf103_Supplementary_Data [file czaf103_supplementary_data.zip › Table 2.docx]

Table 2. Descriptive analysis of the mean OOPE for 2021 with the covariates considered, among individuals who reported any expenditure.

|  | Total OOPE  (Mean) | 6,433 observations in 2021  (%) |
| --- | --- | --- |
| SS stability |  | |
| Stable | 1,405.13 | 48.75 |
| Unstable with SS | 1,375.03 | 13.48 |
| Unstable without SS | 1,152.31 | 7.38 |
| Without SS | 1,163.16 | 30.39 |
| Age |  | |
| 50-59 | 1,049.84 | 9.41 |
| 60-69 | 1,142.32 | 35.67 |
| 70-79 | 1,437.80 | 36.56 |
| 80 and more | 1,506.34 | 18.36 |
| Sex |  | |
| Men | 1,372.35 | 37.51 |
| Women | 1,270.77 | 62.49 |
| Years of school |  | |
| 0 | 1,122.71 | 16.06 |
| 1-6 | 1,280.67 | 51.78 |
| 7-12 | 1,290.04 | 22.48 |
| 13-18 | 1,802.34 | 9.01 |
| 19 and more | 1,943.02 | 0.67 |
| Marital status |  | |
| Married or inunion | 1,345.82 | 58.84 |
| Single | 1,256.05 | 41.16 |
| Employment status |  | |
| Working | 994.01 | 25.88 |
| Not working | 1,418.82 | 74.12 |
| Economic situation* |  | |
| Good | 1,409.83 | 31.97 |
| Fair | 1,114.80 | 61.41 |
| Poor | 1,120.49 | 6.62 |
| Locality size |  | |
| >100,000 inhabitants | 1,402.67 | 54.00 |
| <100,000 inhabitants | 1,198.74 | 46.00 |
| Morbidity |  | |
| 0 | 1,002.15 | 28.01 |
| 1 | 1,240.41 | 37.40 |
| 2 | 1,631.31 | 34.59 |
| Disability |  | |
| No | 1,114.41 | 66.78 |
| Yes | 1,701.52 | 33.22 |
| Smoking status |  | |
| No | 1,334.10 | 91.71 |
| Yes | 1,029.63 | 8.29 |
| Self-rated health status^ |  | |
| Good | 1,056.35 | 31.61 |
| Fair | 1,189.09 | 57.91 |
| Poor | 1,793.46 | 10.48 |

* The sample is 5,786 observations because not all participants answered this question.

^ The sample is 5,802 observations because not all participants answered this question.

SS- Social security.
