# Supplementary material for: Out-of-pocket healthcare expenditures in older Mexican people based on their social security status
Source: Health Policy Plan. 2025 Dec 3;41(2):252–61. doi: 10.1093/heapol/czaf103 (PMC12906755; doi:10.1093/heapol/czaf103)
Supplement: czaf103_Supplementary_Data [file czaf103_supplementary_data.zip › Table 3.docx]

Table 3. Quantile regression models for OOPE in 2021.

|  | Model 1  (n= 6,433) | Model 2  (n= 5,768) | Model 3  (n= 5,765) |
| --- | --- | --- | --- |
|  | OR  CI 95% | OR  CI 95% | OR  CI 95% |
| SS stability |  |  |  |
| Stable | Reference | | |
| Unstable with SS | -94.66**  (-149.37 – -39.96) | -30.97  (-77.45 – 15.51) | -35.35  (-71.59 – 0.90) |
| Unstable without SS | -94.66**  (-150.77 – -38.56) | -57.65  (-122.37 – 7.06) | -60.67*  (-118.99 – 2.39) |
| Without SS | -94.66**  (-137.24 – -52.09) | -40.50*  (-79.28 – -1.71) | -45.33*  (-79.06 – 11.62) |
| Age^ |  | 5.15**  (3.18 – 7.13) | 3.24**  (1.47 – 5.02) |
| Sex |  |  |  |
| Men |  | Reference | |
| Women |  | - | - |
| Years of school^ |  | 12.40**  (7.85 – 16.95) | 16.39**  (12.36 – 20.42) |
| Marital status |  |  |  |
| Married or inunion |  | Reference | |
| Single |  | - | - |
| Employment status |  |  |  |
| Working |  | Reference | |
| Not working |  | 119.64**  (87.89 – 151.38) | 67.41**  (38.79 – 96.03) |
| Economic situation |  |  |  |
| Good |  | Reference | |
| Fair |  | -53.44*  (-99.27 – -7.60) | -87.19**  (-132.17 – -42.21) |
| Poor |  | -45.77  (-97.70 – 6.14) | -94.81**  (-147.61 – -42.01) |
| Locality size |  |  |  |
| >100,000 inhabitants |  | Reference | |
| <100,000 inhabitants |  | - | - |
| Morbidity |  |  |  |
| None |  |  | Reference |
| 1 |  |  | 53.55**  (26.39 – 80.70) |
| 2 |  |  | 189.11**  (141.10 – 237.11) |
| Disability |  |  |  |
| No |  |  | Reference |
| Yes |  |  | 110.87**  (68.43 – 153.32) |
| Smoking status |  |  |  |
| No |  |  | Reference |
| Yes |  |  | - |
| Self-rated health status |  |  |  |
| Good |  |  | Reference |
| Fair |  |  | 70.20**  (43.19 – 97.21) |
| Poor |  |  | 275.62**  (171.33 – 379.90) |
| Wald test (p value) | 0.0001 | 0.0000 | 0.0000 |

^ These variables were included as continuous in the regression models.

SS- Social security.

*p <0.05

**p <0.01
